# Nanoscale modification of silicon and germanium surfaces exposed to low-energy helium plasma

**DOI:** 10.1038/s41598-019-46541-w

**Published:** 2019-07-12

**Authors:** Matt Thompson, Luke Magyar, Cormac Corr

**Affiliations:** Research School of Physics and Engineering, Australian National University, Canberra, ACT 2601 Australia

**Keywords:** Synthesis and processing, Surfaces, interfaces and thin films

## Abstract

Complex surface nanostructures were observed in germanium and silicon samples exposed to low energy (24 or 36 eV ion kinetic energy) helium plasma. Pyramidal growth is observed in germanium across the temperature range studied (185 °C to 336 °C), while significant modification in silicon was only observed at 630 °C. Nano-wire growth was observed in both germanium and silicon, and appears to be linked to the strength of the electric field, which in turn determines the implantation energy of the helium ions. Nanostructure formation is proposed to be driven by surface adatom migration which is strongly influenced by an Ehrlich-Schwoebel-type surface instability. The role of helium in this model is to drive germanium interstitial formation by ejecting germanium atoms from lattice sites, leading to germanium interstitial diffusion towards the sample surface and subsequent adatom and surface nanostructure formation.

## Introduction

The ability to engineer material surface properties at the nanoscale is central to much of today’s modern technology and a broad range of emerging technologies including high energy-density lithium-ion batteries^1,2^, anti-reflection solar cell coatings^3,4^ and water splitting catalysts^5,6^. The methods available to fabricate these structures are as diverse as the structures themselves, including chemical vapor deposition and plasma-enhanced chemical vapor deposition, lithography, chemical etching, and many others.

Here an alternative nanostructure fabrication scheme is proposed, which is able to generate a range of different nanoscale morphologies in key technological materials silicon and germanium. If materials are exposed to low energy (~30–70 eV kinetic energy ions) helium plasma at roughly 0.3–0.5 times the melting temperature, complex wire-like nanostructures are known to evolve form the surface^7^. The process has been widely studied in the context of fusion reactor wall materials such as tungsten^8,9^ and has often been associated with the simultaneous development of helium-filled bubbles beneath the material surface^10–12^. This approach towards plasma processing is appealing as it requires no hazardous chemicals and the inert process gas, helium, is not consumed during the process so can be recycled.

The mechanism by which helium drives this nanostructure growth remains an outstanding question. Due to the low helium ion energies involved (30–70 eV) and helium’s low atomic mass the sputtering yield of the target material is expected to be extremely low. Consequently, nanostructure growth must be driven by other means. This is often attributed to changes in the material driven by the formation of sub-surface helium bubbles, either due to changes in the surface elasticity^13^ or migration and subsequent rupture of bubbles as they make contact with the surface^10,11^.

In this work a range of possible nanostructures that can be generated by helium plasma exposure are shown, including dense fields of nano-wires, pyramids, and hemi-spherical surface pits. It is proposed that surface diffusion, in competition with sputtering from impurity ions in the plasma, is the driving mechanism behind the changes observed in silicon and germanium.

## Results

### Surface modification of germanium

**Figure 1:**
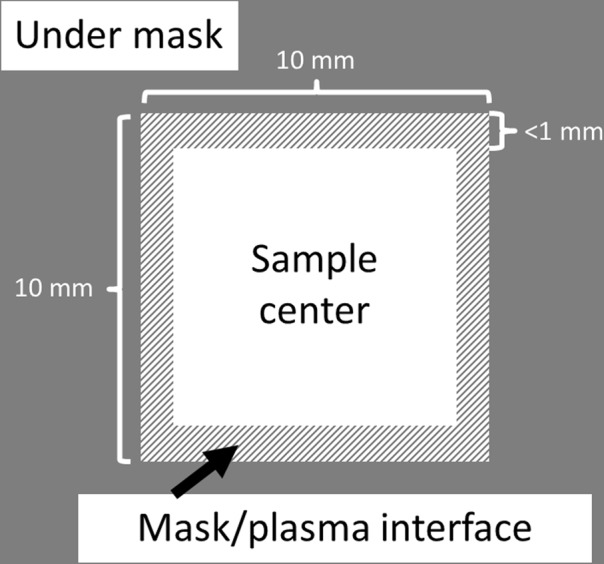
Schematic of the sample mask configuration. The three main regions of interest are near the center of the 10 mm × 10 mm plasma exposed region of the sample, near the plasma/mask interface (<1 mm from the mask edge), and under the mask.

SEM images were taken of germanium samples in three different regions – near the center of the sample, under the mask, and in an area close to the plasma/mask interface. A schematic highlighting these regions is shown in Fig. 1.

**Figure 2:**
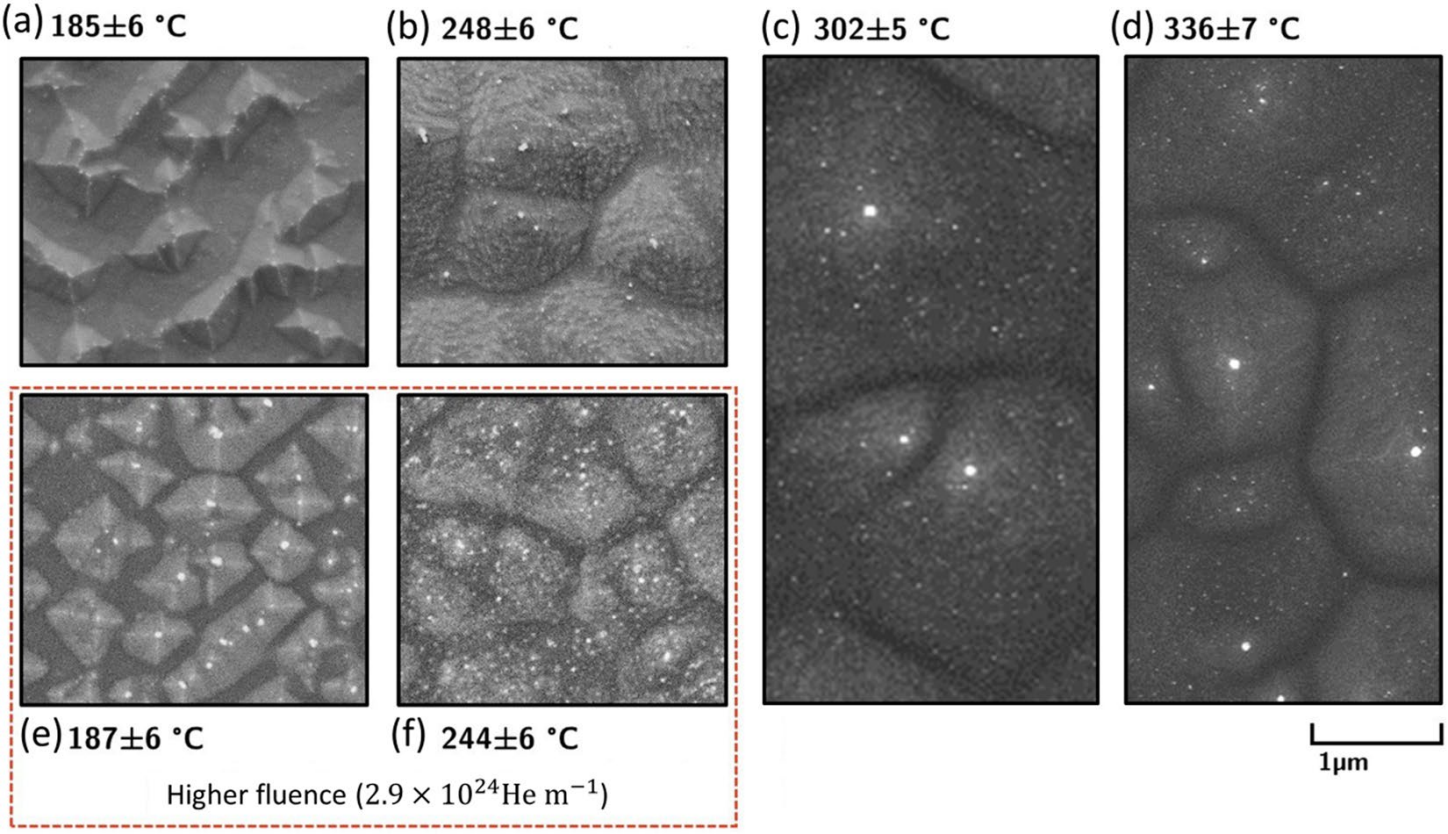
Germanium surfaces exposed to 24 eV helium ions at a fluence of 1.5 × 10^24^ He m^−1^ for exposure temperatures of (**a**) 188 ± 6 °C, (**b**) 248 ± 6 °C, (**c**) 302 ± 5 °C, and (**d**) 336 ± 7 °C. Germanium surfaces exposed to a higher plasma fluence of 2.9 × 10^24^ He m^−1^ are also shown for samples exposed at temperatures of (**e**) 187 ± 6 °C and (**f**) 244 ± 6 °C. Surface structures show strong faceting at 185 ± 6 °C, and become larger and less well-orientated at higher temperatures.

Figure 2 shows the surface features of germanium near the sample center exposed to helium fluences of (Fig. 2a–d) 1.5 × 10^24^ He m^−2^ and (Fig. 2e,f) 2.9 × 10^24^ He m^−2^ for an incident helium ion energy of 24 eV. Here, pyramidal structures can be seen on all surfaces with the structures tending to grow larger with increasing temperature. A rounded peak can be observed on many of the structures. At 185 ± 6 °C pyramids appear to grow outward from an almost flat plane, with pyramidal faces aligned with (111) crystallographic planes. At higher temperatures the alignment of the pyramids’ faces becomes less pronounced. Instead, the faces are covered in rough terraces where the flat surfaces of the terraces show crystallographic alignment. Plasma exposures performed at the higher fluence of 2.9 × 10^24^ He m^−2^ are qualitatively similar to those of the lower fluence of 1.5 × 10^24^ He m^−2^, though the feature density may be somewhat greater at 187 ± 6 °C.

**Figure 3:**
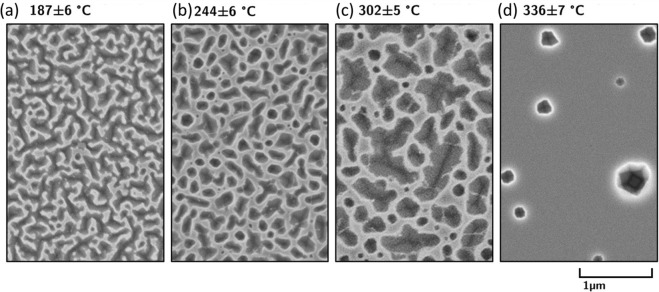
Germanium surfaces covered by the sample holder mask after 24 eV helium ions at a fluence of 1.5 × 10^24^ He m^−1^ for exposure temperatures of (**a**) 185 ± 6 °C, (**b**) 248 ± 6 °C, (**c**) 302 ± 5 °C, and (**d**) 336 ± 7 °C. Surface pitting is observed at all temperatures, with pits growing further from the plasma mask at higher temperatures.

Nanostructures also form beneath the sample mask, despite being sheltered from the plasma (Fig. 3). Here, rather than upward pyramids the features appear to form as downward growing pits, with the size of these structures growing larger with increasing temperature. Between 302 ± 5 °C and 336 ± 7 °C there is a qualitative difference in the growth characteristics of the pits, with dense, interconnected pits forming at 302 ± 5 °C and sparse but large holes forming at 336 ± 7 °C. These features cannot be explained by direct interaction with the plasma itself, so are instead thought to form due to vacancy (or helium-vacancy cluster) migration through the bulk from the plasma exposed region. Similar pits are known to be produced by vacancy clustering in germanium crystals grown via the Czochralski process^14^. The pits at 336 ± 7 °C extend much further away from the mask edge than the features observed at lower temperatures, up to a distance of several millimeters. The qualitative shift in behavior may therefore be due to a greater vacancy diffusion distance at higher temperatures leading to more dispersed features, rather than a reduction in the vacancy flux.

**Figure 4:**
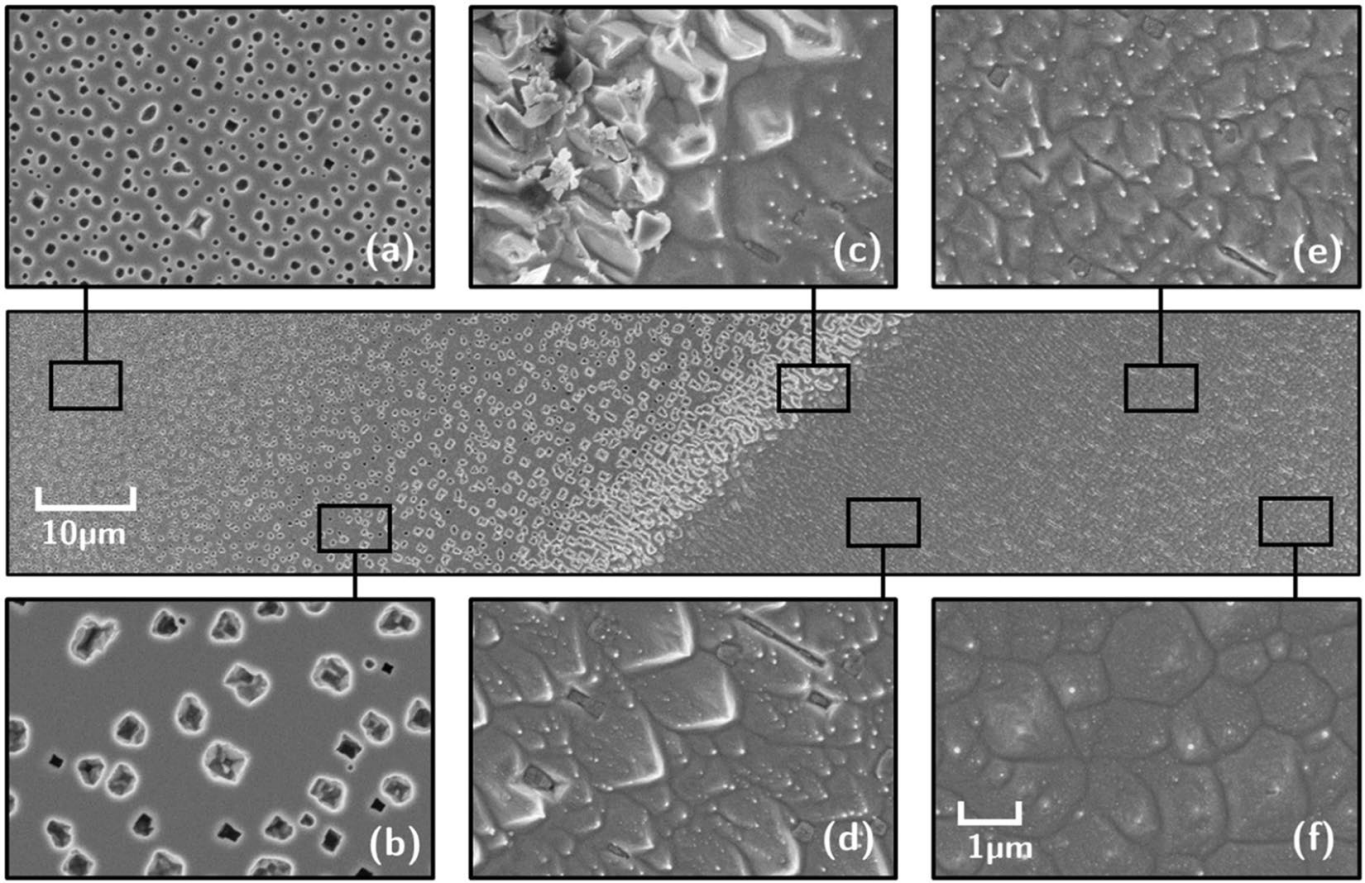
The transition region near the sample holder mask for the germanium temperature exposed to 24 eV helium ions at 336 ± 7 °C. The central image shows a wide view of the transition region, while the inserts (**a**–**f**) show zoomed in images of areas at different distances to the mask position. The mask position itself corresponds to image (**c**).

In the intermediate region around the mask edge (Fig. 4, example for 336 ± 7 °C) a transition region is observed between the pits in the masked region and the pyramidal structures in the exposed region. Near the mask edge itself pyramids appear to point away from the mask rather than upright. This is likely the result of the presence of stronger electric fields in the vicinity of the (highly conductive) tantalum mask which influences the direction of the plasma flow and increases the implantation energy of the helium ions. In this region the pyramids appear to point into the plasma flow which could be explained by sputtering from trace impurity species within the plasma.

**Figure 5:**
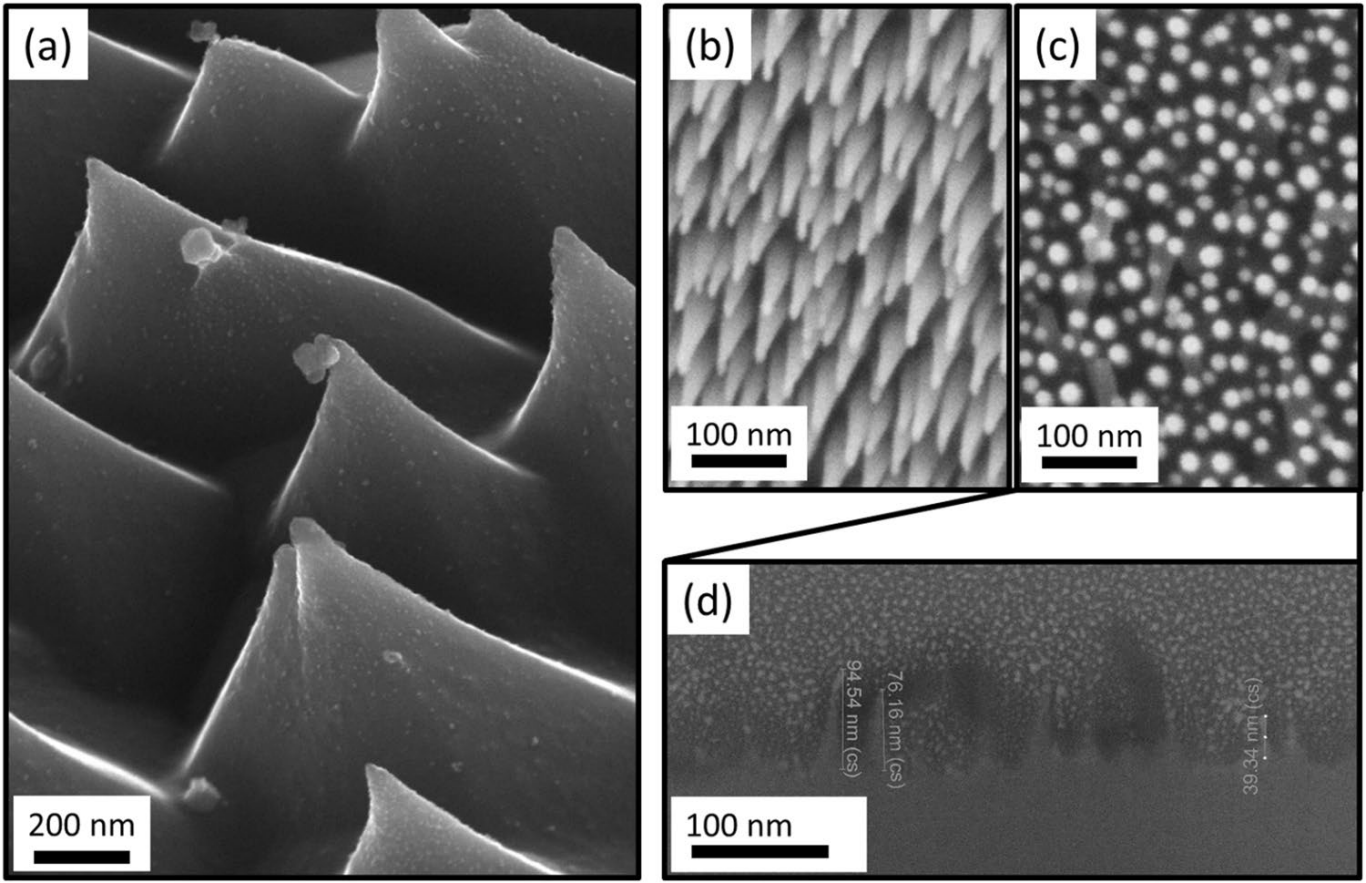
(**a**) Well-formed pyramids growing in the vicinity of the sample holder mask for the germanium sample exposed to 24 eV helium ions at 336 ± 7 °C. Images for an additional germanium sample exposed to 36 eV helium ions at 240 ± 10 °C (**b**) near the sample holder mask and (**c**) in the sample center. (**d**) Cross-sectional image of the region shown in (**c**).

Figure 5a shows a dense field of well-formed pyramidal structures that were observed near one of the mask edges of the 336 ± 7 °C exposure temperature germanium sample. Features of this specific morphology were not observed near other mask edges. To further demonstrate this effect, SEM images (Fig. 5b,c) and an FIB cross-sectional image (Fig. 5d) are shown for a germanium sample exposed to helium plasma at a temperature of 240 ± 10 °C and a higher helium ion energy of 36 eV. Near the sample mask (Fig. 5b) this new sample shows much finer cone-like structures than observed for the lower electron temperature plasma. Furthermore, in the center of the sample (Fig. 5c) very fine wire-like structures with ~10 nm diameters were observed which covered most of the sample surface. FIB cross-sections taken in this central position (Fig. 5d) reveal nano-wire lengths up to approximately 100 nm.

**Figure 6:**
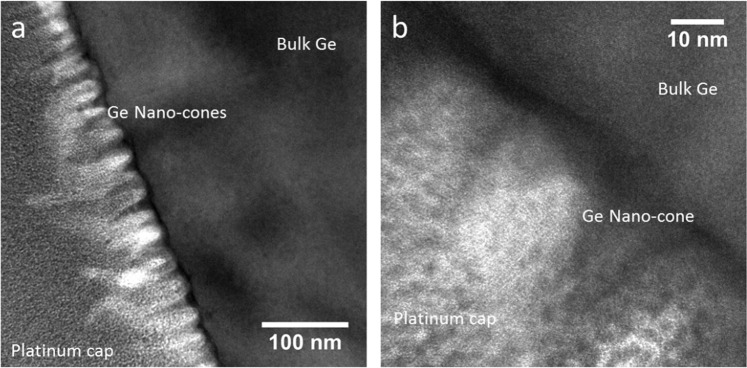
(**a**,**b**) Transmission electron micrographs of germanium exposed to 36 eV helium ions at 240 ± 10 °C in an upstream position within the MAGPIE linear plasma device at different levels of magnification. A dense layer of nano-structures with a conical or wire-like shape cover the sample surface. Close inspection of (**b**) reveals crystallographic alignment between nano-structures and substrate material. Nano-scale bubbles are not observed below the sample surface.

Closer inspection of the sample exposed to helium plasma at 240 ± 10 °C for 36 eV helium was conducted using a JEOL 2100F field emission gun Transmission Electron Microscope (TEM), as shown in Fig. 6. TEM shows dense surface coverage of the germanium sample with conical or wire-like nanostructures. No other structures are observed below the sample surface. This behavior is distinctly different to the case with metals where nanoscale bubbles are known to form beneath the surface wires^8^. To determine whether impurity deposition could have influenced nano-wire growth energy dispersive X-ray spectroscopy was performed via the TEM. Tantalum was not detected, eliminating the possibility of erosion and re-deposition from the sample holder mask influencing nanowire growth. The only major impurity signals were platinum, copper, carbon, and iron. These elements were not associated with the surface nanostructures as platinum was deposited on the surface for FIB cross-section preparation, copper and carbon originated from the TEM specimen grid, and iron is likely to have originated as a background signal from the TEM column itself. Impurity deposition can therefore be eliminated as a significant mechanism driving nanostructure formation.

This observation of different qualitative behavior between the 36 eV helium (240 ± 10 °C) and 24 eV helium (248 ± 6 °C) exposures emphasize the importance of the plasma itself in driving the surface changes observed. These differences indicate that the nanostructures form as a result of a dynamic equilibrium between plasma-driven nanostructure growth and temperature-driven surface relaxation. The exact reason for this difference has not yet been determined, but it could be related to a reduction in the proportion of helium that is reflected from the sample surface at higher implantation energies^15^, which in turn would increase the equilibrium concentration of helium within the material during irradiation.

### Surface modification of silicon

**Figure 7:**
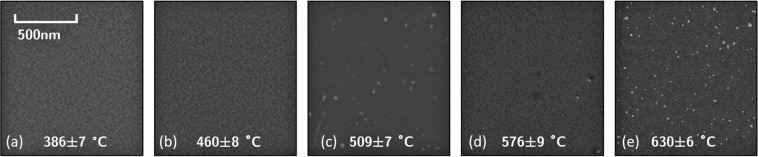
Silicon surfaces exposed to 24 eV helium ions at a fluence of 1.5 × 10^24^ He m^−1^ for exposure temperatures of (**a**) 386 ± 7 °C, (**b**) 460 ± 8 °C, (**c**) 509 ± 7 °C, (**d**) 576 ± 7 °C, and (**e**) 630 ± 7 °C. Limited nanostructure formation is observed in this region of the sample at any exposure temperature.

SEM images of silicon near the center of the sample (Fig. 7) reveal much less pronounced nanostructure formation than was observed for germanium. No surface changes were observed at all for samples exposed to 24 eV helium ions below 460 °C, while beyond 500 °C features were sparse and surface coverage uneven. No changes were observed at all beneath the mask.

**Figure 8:**
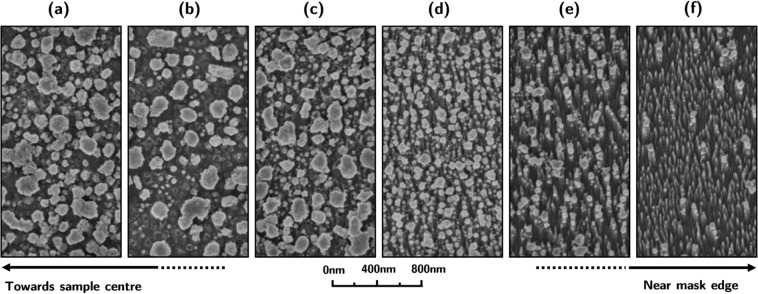
Silicon surfaces exposed to 24 eV helium ions at 630 ± 6 °C in a region near the sample mask. Figure shows a progression from the types of nanostructures that formed in that region closer to the sample center (**a**), in intermediate positions (**b**–**e**), and closer to the mask (**f**).

**Figure 9:**
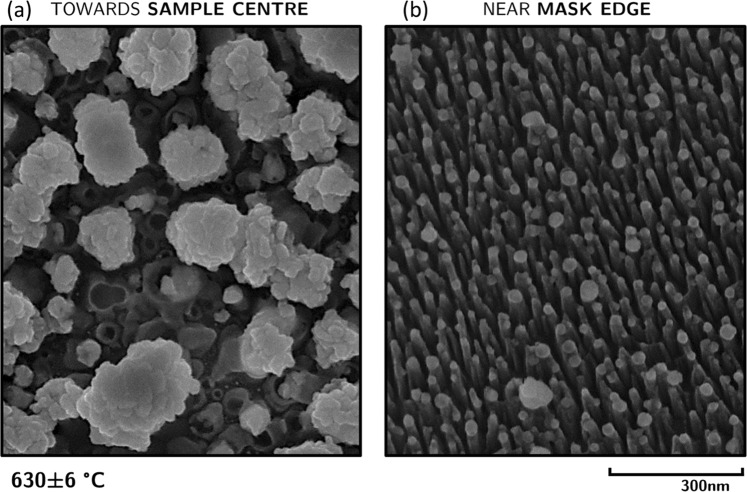
A close up image of the features shown in Fig. 8 for (**a**) dust-like particles and (**b**) nano-wires.

**Figure 10:**
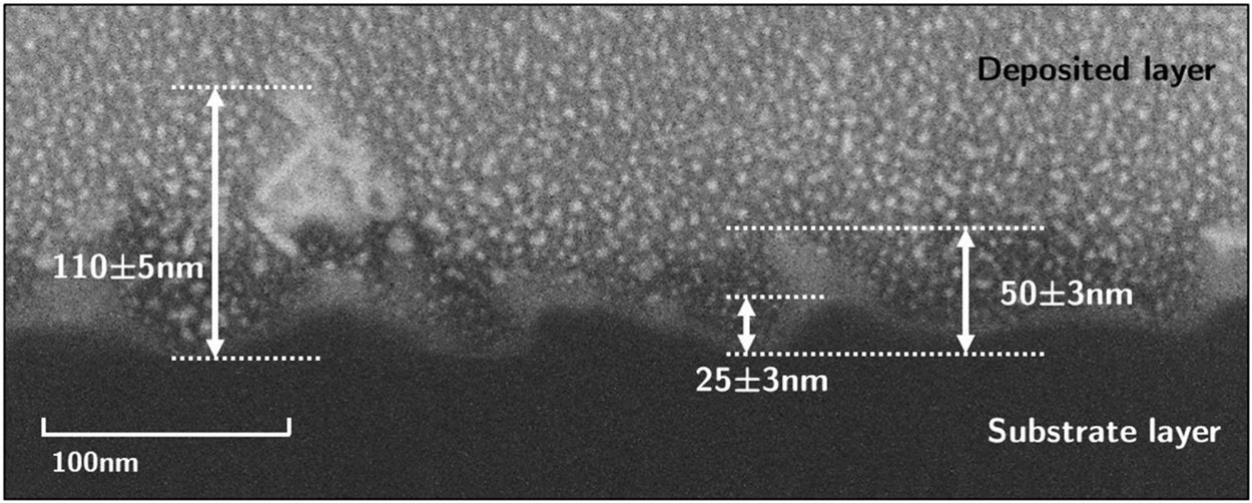
FIB cross-section of the nano-wires shown in Fig. 9b. Nanowires appear to grow above a rippled surface, with wires facing into the direction of the plasma flow.

For 24 eV helium ion exposure at 630 ± 6 °C significant surface modification was observed in the exposed region in the immediate vicinity of the sample mask (Fig. 8). On the side of this region closer to the sample center dust-like particles are observed. As the mask is approached, these dust-like features are replaced by a dense field of fine nano-wires. Closer inspection of these features (Fig. 9) reveals a diameter of approximately 100–300 nm for the dust particles and ~10 nm for the nano-wires. A FIB cross-section of the nano-wire structures is shown in Fig. 10. The nano-wires appear to grow from the peaks of a ripple-like structure and point away from the sample mask. The difference in behavior in the vicinity of the sample mask is again believed to be due to stronger electric fields expected in this region, which would result in a higher helium implantation energy. TEM was performed on the sample exposed to helium plasma at 630 ± 6 °C to determine whether nanoscale bubbles were present (not shown), though no sub-surface structures of any kind were found.

## Discussion

The absence of sub-surface nano-bubbles in this work is difficult to reconcile with the popular explanation of Kajita *et al*.^8^ which proposes surface nanowires in tungsten are produced by a process of repeated surface deformation due to near-surface helium-filled bubbles as they interact with the surface. A different mechanism is therefore necessary to explain how these extended structures form in silicon/germanium. Similar cone-like structures have been observed by Nishijima *et al*.^16^ in chromium and beryllium within several different linear plasma devices which was attributed primarily to impurity deposition from the sample holder masks, whereby conical structures are produced by preferential sputtering of areas without impurity deposition. However, as impurity deposition was not observed in the present work this cannot be the driving force behind the nanostructures observed here. Indeed, Nishijima *et al*. note themselves that their mechanism cannot account for all experimental observations, such as nano-cone formation in beryllium exposed to helium plasma in the PISCES-B linear plasma device in the absence of impurity sources^17^. An alternative explanation for nanostructure formation growth under helium plasma is provided by Kajita *et al*.^18^, who discovered an enhanced nanowire growth regime where tungsten ions were deposited on a tungsten substrate after sputtering from a wire upstream within the helium plasma. Millimeter-length nanowires were generated in this manner, which was attributed to deposition of surface adatoms onto the tungsten substrate.

Here, we expand on Kajita *et al*.^18^ view by proposing that in the general case nanostructure formation is a peculiar form of homoepitaxial growth where instead of depositing surface adatoms form the plasma surface adatoms are generated via helium-material interactions. Viewing surface island growth through the lens of epitaxy is advantageous as this is a mature, widely studied field where many different growth regimes have already been identified which can produce diverse surface structures including ordered nanowires^19^, nanorods^20^, surface islands^21^, and surface layers with different degrees of roughness^22^.

The most interesting features observed in this work, the extended surface nanowires, are formed over a relatively narrow temperature range in both germanium and silicon with optimal temperatures that differ between materials. This behavior can readily be explained in terms of surface adatom behavior. If surface adatoms are introduced randomly to a material surface they will naturally cluster in order to minimize the surface energy. Over time atomic terraces will form. Adatoms that are placed on top of an atomic terrace will encounter a larger potential barrier in order to migrate towards a lower terrace than ordinary surface diffusion (the Ehrlich-Schwoebel diffusion batter), leading to net adatom migration proportional to surface curvature away from regions of positive curvature towards regions of negative curvature^23^ (i.e. away from valleys and towards mounds). This mechanism naturally leads to a narrow temperature band between energies required to facilitate surface adatom migration, and those required to overcome the Ehrlich-Schwoebel diffusion barrier and counteract the diffusion bias. As sputtering is negligible under low energy helium plasma these surface instabilities are able to grow without limit.

For this model to make sense a source of surface adatoms is required. The most probable source of surface adatoms is due to interactions between implanted helium atoms and the target material. Helium is implanted into the sample at low energies (20–40 eV) during plasma irradiation. Due to low ion energies involved knock-on damage is not expected to occur. Molecular dynamics simulations have suggested interstitial helium is able to cluster in tungsten and create vacancy-type defects via the ejection of a tungsten atom form the matrix^24^. If a similar process were to occur in germanium, the incident helium ions from the plasma could potentially drive significant vacancy formation near the sample surface. Helium would then stabilize the resulting vacancy, preventing vacancy-interstitial recombination. The germanium atoms that are ejected from their lattice positions take interstitial positions and diffuse until they reach the sample surface. Upon reaching the surface they become surface adatoms. Due to the constant influx of helium and subsequent creation of surface adatoms these surface adatoms are not in thermal equilibrium with the surface itself so drive surface island growth via clustering. This would lead to the formation of surface instabilities analogous to those described by the Ehrlich-Schwoebel instability model of Rusponi *et al*.^23^, which drives net adatom diffusion towards regions of negative curvature (i.e. the peaks of nanostructures). At low temperatures nanostructures do not form as there is not enough energy to drive surface adatom diffusion, inhibiting island growth. At higher temperatures adatoms are able to overcome the Ehrlich-Schwoebel energy barriers, leading to first the formation of larger surface features, and later surface recovery towards favored crystallographic orientations via back-diffusion of adatoms once the diffusion barriers can be overcome^23^. Nanostructure formation would be enhanced by increasing the helium flux, or by increasing the incident helium energy to reduce the fraction of helium ions that are reflected from the surface at these low energies^15,25^.

As no bubble formation is observed in silicon or germanium, vacancies are likely to eventually recombine with the surface as well where they will preferentially drive surface pit formation^26^, enhancing surface roughening further. Germanium is an unusual case as its vacancy diffusivity is exceptionally high^14^: roughly 2 × 10^−7^ cm^2^/s at 240 °C, similar to that of the self-diffusion of a body-centered cubic metal at its melting point. This high vacancy self-diffusion rate could account for diffusion of hundreds of microns to millimeters over the temperatures studied and may account for the formation of pits outside the plasma exposed region (as shown in Fig. 3). This is evidence that vacancy and interstitial separation must occur.

Developing this mechanism further into a quantitative model of surface nanowire growth will be the subject of future work. Further work will also be required to establish the degree of control that can be achieved through helium plasma driven modification of material surfaces. Substrate temperature and incident ion energy appear to be important factors, however it is not yet clear whether these can be manipulated to modify growth properties in a precise or consistent way.

## Conclusion

Complex surface nanostructures were observed in germanium and silicon samples exposed to low energy (24–36 eV kinetic energy ions) helium plasma. Pyramidal growth is observed in germanium across the temperature range studied (185 °C to 336 °C) for 24 eV helium exposure, while significant modification in silicon was only observed in a limited region near the sample holder mask at 630 °C. Nano-wire growth was observed in both germanium and silicon, and appears to be linked to the strength of the electric field, which in turn determines the implantation energy of the helium ions.

Nanostructure formation is proposed to be driven by surface adatom migration via an Ehrlich-Schwoebel-type surface instability. The role of helium in this model is to drive germanium interstitial formation by ejecting germanium atoms from lattice sites, suppressing vacancy-interstitial recombination and driving adatom and surface nanostructure formation. A similar process is likely to explain behavior of nanostructures observed in other materials such as silicon, beryllium and tungsten, where differences can be explained by differences in self-interstitial and vacancy kinetics.

Helium-driven nanowire growth may be useful in applications that require high surface area (photo-catalysis) and/or low reflectivity (solar panel surface processing), or in applications where the nanoscale nature of the structures is important for device performance such as high energy-density silicon/germanium lithium-ion battery anodes, where small wire diameters reduce degradation over multiple charge/discharge cycles^27,28^.

## Methodology

P-type silicon (boron doped, 0.007–0.005 Ω cm^−1^) and germanium (gallium doped, 0.1–0.35 Ω cm^−1^) samples were exposed to helium plasma at the Australian National University’s MAPIE linear plasma device facility^29^ to a plasma fluence of 1.5 × 10^24^ He ions m^−2^ with an approximate helium ion incident energy of either 24 eV or 36 eV. Incident helium ion energies were determined by calculating the plasma sheath potential from the I-V characteristic of a Langmuir single probe. As the electron temperatures are expected to be much higher than ion temperatures acceleration of singly charged helium across the plasma sheath dominates helium implantation energies. Helium ion energies were approximately constant for any given irradiation. Doped materials were used to increase the electrical conductivity of the target. To determine the effect material temperature has on nanostructure formation several samples of silicon and germanium were exposed at temperatures ranging from 185 °C to 336 °C for germanium samples, and 386 °C to 630 °C for silicon. During plasma exposure samples were held at a constant temperature which was maintained by both varying the current through a heating element that was built into the sample holder, and by varying the plasma duty cycle. The temperature was measured via a thermocouple that was inserted into the back of the sample holder. During plasma exposure samples were attached to the sample holder via a spring-loaded tantalum mask. The presence of this mask allowed comparative measurements to be made between regions that were directly exposed to helium plasma and regions that were covered by the mask.

After plasma exposure the sample surface morphology was measured via scanning electron microscopy (SEM, Zeiss UltraPlus analytical FESEM) to qualitatively identify how surface structures varied with material temperature during plasma exposure. Focused ion beam (FIB, FEI Helios 600 NanoLab) cross sections were performed on selected samples to perform a more detailed characterization of surface nanostructures and to search for sub-surface features.

Transmission Electron Microscope (TEM) specimens were prepared using the FEI Helios 600 NanoLab FIB used for SEM cross-section preparation. TEM measurements were performed on a JEOL 2100F field emission gun TEM.
